# Therapy of Chagas Disease: Implications for Levels of Prevention

**DOI:** 10.1155/2012/292138

**Published:** 2012-03-05

**Authors:** Sergio Sosa-Estani, Lisandro Colantonio, Elsa Leonor Segura

**Affiliations:** ^1^Instituto Nacional de Parasitología “Dr. Mario Fatala Chaben” y Centro Nacional de Diagnóstico e Investigación de Endemo-epidemias (CeNDIE), ANLIS “Dr. Carlos G. Malbrán”, Ministerio de Salud, Avendia Paseo Colón 568, 1063 Buenos Aires, Argentina; ^2^Consejo Nacional de Investigaciones Científicas y Técnicas (CONICET), Avendia Rivadavia 1917, 1033 Buenos Aires, Argentina; ^3^Instituto de Efectividad Clínica y Sanitaria (IECS), Dr. Emilio Ravignani 2024, 1414 Buenos Aires, Argentina; ^4^Departamento de Salud Pública, Facultad de Medicina, Universidad de Buenos Aires, Marcelo T. de Alvear 2202, 1121 Buenos Aires, Argentina

## Abstract

This paper reviews the evidence supporting the use of etiological treatment for Chagas disease that has changed the standard of care for patients with *Trypanosoma cruzi* infection in the last decades. Implications of this evidence on different levels of prevention as well as gaps in current knowledge are also discussed. In this regard, etiological treatment has shown to be beneficial as an intervention for secondary prevention to successfully cure the infection or to delay, reduce, or prevent the progression to disease, and as primary disease prevention by breaking the chain of transmission. Timely diagnosis during initial stages would allow for the prescription of appropriate therapies mainly in the primary health care system thus improving chances for a better quality of life. Based on current evidence, etiological treatment has to be considered as an essential public health strategy useful to reduce disease burden and to eliminate Chagas disease altogether.

## 1. Introduction

One hundred years after Carlos Chagas identified and described the *Trypanosoma cruzi* (*T. cruzi)* infection, there are still millions of infected people and thousands of newly diagnosed cases each year with Chagas disease (CD). The scientific community has intermittently increased the knowledge and understanding of how to manage patients with acute and chronic CD [1]. Nonetheless, much more research is still needed in order to improve care and answer many unknown questions regarding this debilitating and widespread disease, which has been estimated to affect about 8 million chronically infected people just in the Americas [2]. 

The goal of etiological treatment against Chagas disease is to eliminate the parasite (*T. cruzi*) from the infected individual, to decrease the probability of developing clinical manifestations of the disease (e.g., cardiovascular or digestive diseases), and to break the chain of disease transmission [3].

Currently, there is a new scenario regarding the recommended etiological treatment against *T. cruzi* infection. It is based on several strong evidences supported by basic research, clinical trials, observational studies, and expert opinions. In this paper, we review the current evidence supporting etiological Chagas disease therapy organized according to different levels of prevention. Additionally, we discuss the tools available to demonstrate cure in these patients, and the need for further research required to improve care for *T. cruzi *infected people. 

## 2. Materials and Methods

We reviewed the evidence supporting the use of anti-*T. cruzi *pharmacotherapy (etiological treatment) in order to reduce or avoid the morbidity and mortality of Chagas disease applied on different levels of prevention. In this regard, a MEDLINE search was conducted from January to July 2011, using the term “Chagas disease” with the subheadings “diagnosis,” “prognosis,” “treatment,” drug names (nifurtimox, benznidazole, and other drugs), “clinical trials,” and “observational studies.” No restrictions regarding year, language or country of publication were applied. Recent guidelines as well as ongoing and unpublished studies were also identified by consulting researchers and experts in the field. Evidence was organized according to the levels of prevention addressed by the retrieved guidelines or epidemiological researches. Finally, we reviewed the strength of evidence for each indication in each level of prevention.

### 2.1. Definitions

#### 2.1.1. Levels of Prevention

Leavell and Clark's have defined three different levels of prevention in human health (primary, secondary, and tertiary) in a classical textbook published in 1953 [4]. Each of them includes different means of intervention according to the natural history of the disease.

**Primary Prevention**

These strategies intend to avoid the disease's development, including the acquisition of new infection. Most population-based health promotion activities are primary preventive measures.

**Secondary Prevention**

These strategies attempt to diagnose and treat an existing disease in its early stages before it results in significant morbidity.

**Tertiary Prevention**

These treatments aim to reduce the negative impact of established disease by restoring function and reducing disease-related complications.

In the last decades, Jamoulle has proposed a fourth concept (quaternary prevention), which was incorporated by the WONCA International Classification Committee [5]. In this regard, quaternary prevention describes the set of health activities aimed to mitigate or avoid the consequences of unnecessary or excessive interventions in the health system.

Strength of recommendations as well as the quality level of the evidence supporting these recommendations were addressed according to the Quality Standards Subcommittee or the Clinical Affairs Committee of the Infectious Diseases Society of America (IDSA) [6].

### 2.2. Strength of the Recommendation

- Both strong evidence for efficacy and substantial clinical benefit support recommendation for use. It should always be offered.
- Moderate evidence for efficacy—or strong evidence for efficacy but only limited clinical benefit—support recommendation for use. It should generally be offered.
- Evidence for efficacy is insufficient to support a recommendation for or against use. Or evidence for efficacy might not outweigh adverse consequences (e.g., drug toxicity, drug interactions) or cost of the treatment under consideration: optional.
- Moderate evidence for lack of efficacy or for adverse outcome supports a recommendation against use. It should generally not be offered.
- Good evidence for lack of efficacy or for adverse outcome supports a recommendation against use. It should never be offered.

### 2.3. Quality of Evidence Supporting the Recommendation

Type I: evidence from at least one properly designed randomized, controlled trial.
Type II: evidence from at least one well-designed clinical trial without randomization, from cohort or case-controlled analytic studies (preferably from more than one center), or from multiple time-series studies, or dramatic results from uncontrolled experiments.
Type III: evidence from opinions of respected authorities based on clinical experience, descriptive studies, or reports of expert committees.

## 3. Results

### 3.1. Recommendations of Therapy and Strength of Evidence

Several papers and guidelines have been published in the last years [7–40], supporting with different levels of strength that etiological treatment is an effective intervention on both the individual and public health. These studies reached levels of evidence ranged from I to III, providing strength of recommendations (A), (B), and (C) (see Table 1). We discuss this criteria applied in different scenarios as follows.

#### 3.1.1. Efficacy of Treatment during Acute Phase of Infection

Several studies have shown the benefit of treatment during acute phase with both benznidazole and nifurtimox with a level of evidence Type I or II [10–15]. The assessment of failure and/or efficacy of treatment on patients treated during acute phase is demonstrable in short time because the parasitemia, whether direct or not (parasitological test or molecular test), becomes negative a few days after the end of treatment. In addition, antibodies disappear completely (seronegativization) in at least 65% of cases, with some studies demonstrating seronegativity in 100% of cases up to 18 months of follow up after treatment. This effect is independent of the age of the patients, including newborns (congenital transmission), children, and adults. The absence of parasitemia demonstrated by direct method such us Strout or micromethod always precedes the reduction of antibodies [10–15].

In general, treatment is well tolerated during the acute phase, and the risk of potential adverse events is counterbalanced by the reduction of clinical manifestations of the acute phase of Chagas infection, and even the associated risk of death. There is wide consensus that all patients undergoing the acute phase of infection or reactivation of chronic infection must be treated (strength of recommendation (A)).

#### 3.1.2. Efficacy of Treatment during Chronic Phase of Infection

Several studies (evidence Type I) have provided support to the use of etiological treatment (benznidazole) during early stages of chronic infection in children [16–22]. Two studies have shown efficacy in this population through double blinded placebo-controlled trials of benznidazole for children aged 6 to 12 years old with asymptomatic *T. cruzi* infection demonstrated approximately 60% efficacy, as assessed by conversion from positive to negative serology results 3 to 4 years after treatment [16, 17]. Rates of seronegativization up to 70% were established with etiological treatment after long-term follow-up (15 years) in South America, and up to 50% after short-term followup (3 years) in Central America. Furthermore, additional studies (evidence Type II) have shown that seronegativization with etiological treatment is also possible in later stages of the chronic infections in adults [7, 18, 23–31]. However, rate of seronegativization of antibodies (serological test) seems to be directly related to the age of patients. Although a complete seronegativization can be obtained in more than 70% of the cases in children, the seronegativization rate have reached about 30% in adult patients after a long-term followup, around 20 years [7, 16–31]. 

Fall in antibodies titers after treatment in children is faster than in adults, even if it does not cross the cutoff to become nonreactive. The decrease in antibody titers is significant during the early months following treatment. A statistically significant reduction is visible at 3 months with EIA and IFA, and at 6 months with IHA [16, 17]. This phenomena was also observed in adult patients [26]. Young children with longer-term followup have higher rates of seronegativization after treatment as compared to child patients with short-term followup, and equal phenomena occurs among adult patients with long-term followup in comparison with adult patients with short followup [9].

Assessment of etiological treatment effect is another important issue under research on Chagas disease. Demonstration of antiparasitic effects after treatment can be performed by the detection of antibodies, parasites, and/or parasite DNA. The success of the treatment is determined by the disappearance of antibodies using serological tests, while therapeutic failure only can be demonstrated by showing the persistence of the parasite using parasitological methods.

The assessment of failure of treatment on patients treated during the chronic phase could be demonstrable in short time because the parasitemia (when it is present by parasitological test or molecular test) disappears at the end of treatment if treatment is successful. When failure occurs, evidence of parasitemia remains positive after treatment (not more than 5% in children or 10% in adults) [9, 10, 17, 18, 20, 41, 42]. However, Gallerano and Sosa [43] showed a higher rate of xeno positives including treatment with nifurtimox, benznidazole, and allopurinol. Though, this last drug (allopurinol) did not show consistent results when they were tested in clinical trials [44, 45].

Other methodologies to evaluate the effectiveness of antiparasitic treatments have been tested, but have not reached consensus to change current testing strategy [9]. Methods to detect the parasite's genomic fragments in tissues and body fluids using polymerase chain reaction (PCR) have proved to be promising tools for the assessment of therapy [13, 36, 45–49], and it was recently standardized for diagnosis [50]. Projects for standardization of PCR for assessing therapy which look for the presence of parasites in the blood are underway. There is agreement that, even with limitation, it will be a useful tool to improve the assessment of treatment failure.

Regarding assessment of treatment efficacy, several researchers are looking for solutions. Molecular methods are showing attributes for making a timely diagnosis at birth [11, 13, 51].

Since tolerance to etiological treatment in children is better than that in adults (see Section 3.3), there is a general agreement that children and adolescents undergoing chronic Chagas phase have to be treated (strength of recommendation (A)). On the other hand, the rate of seronegativization in adult patients (about 30%) based on evidence from observational studies linking the seronegativization to the prevention of clinical disease is currently under research [7, 18, 23–31]. Furthermore, higher rates of adverse events (with a 17% of abandon rate) are seen in adult patients in comparison with children, making this recommendation weaker in adults (strength of recommendation (B)).

In the case of adult patients undergoing the chronic phase of infection, treatment could be offered to them after carefully addressing possible benefits and adverse events. If accepted, therapy should be prescribed due to the strength of evidence available today.

#### 3.1.3. Efficacy of Treatment in Special Cases

Evidence Type III supports that health workers, researchers, and so forth who suffer accidents with infected blood have to be treated under specific protocols [33]. Regarding immunocompromised patients, available studies (evidence Type II) have shown that after etiological treatment, patients recover from severe manifestations of reactivation such as meningoencephalitis, myocarditis, and panniculitis [36, 52]. However, in these cases the main objective is recovery from life-threatening acute events rather than seronegativization, due to the limited ability to interpret serological test results in states of immunosuppression. Since the severity of reactivation and the risk of death are associated, there is a general agreement that these patients must be treated (strength of recommendation (A)). On the other hand, no current evidence supports the use of etiological treatment as prophylaxis in immunocompromised patients with chronic chagasic infection without evidence of reactivation.

Although some studies have reported the etiologic treatment of pregnant women without adverse effects in the new born [32, 33], treatment using benznidazole or nifurtimox is currently not recommended for pregnant women (absolute contraindication) [7, 9]. Additional contraindications to use of etiological treatment include patients undergoing severe acute or chronic liver or kidney disease not related to *T. cruzi* infection (relative contraindication), and lactation (relative contraindication) [7, 9].

Table 1 shows a summary of different scenarios for etiologic treatment against *T. cruzi* infection, and results of different ways for assessing therapeutic response.

### 3.2. Tolerance and Adherence

**Figure 1:**
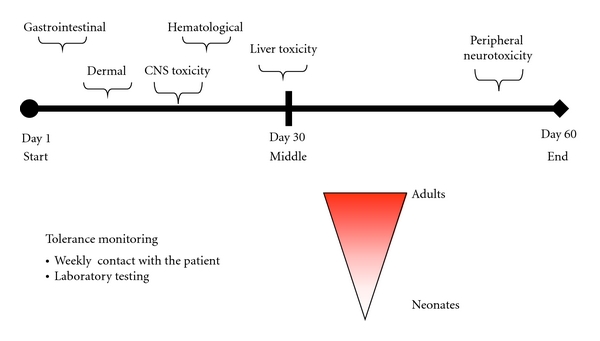
Timeline of side effects of benznidazole and nifurtimox.

During treatment, patients must be under continuous medical supervision. Based on prior experiences, treatment tolerance is good and patients have not denoted serious side effects [8, 19, 27, 41, 53, 54]. Although cases with severe side effects have been reported, they have generally been associated with difficulties in seeking timely medical attention or receiving adequate care. Side effects are more frequently observed in adolescents and adults than in children and babies. In neonates and in children up to 4 years old, tolerance is excellent. In all cases, side effects disappeared when the dose was decreased or the treatment suspended. Types of side effects seen and their distribution during treatment are shown in Figure 1.

 Other types of side effects include reversible clastogenesis and mutagenesis with benznidazole and nifurtimox without any associated manifestations [55, 56], toxicity against other tissues [57] or increased risk of lymphomas in experimental animals [58] have been described, but never demonstrated among a general population of infected patients undergoing treatment [59] and never played a role in animal models [60]. Adequate management of side effects is necessary to carry out treatment as well as to diminish unfounded fears with the use of trypanocidal drugs [53, 61].

### 3.3. Role of Etiological Treatment against T. cruzi Infection on Several Levels of Prevention in Public Health

The recommendations of etiological treatment allow for action on several levels of public health prevention.

Retrieved studies provide evidence for applying strategies of health care to control programs in several countries, whereby a greater portion of the population could get diagnosis, treatment, and cure, generating a new scenario for the reduction in disease burden in the future.

#### 3.3.1. Primary Prevention Level

If the goal is to avoid the acquisition of a new infection, etiological treatment could have an indirect effect when children and young people are treated. Curing children and women in reproductive age would avoid future events of congenital *T. cruzi* transmission in newborns [62] (recommendation (B), and evidence Type III). In addition, the availability of potential blood and organ donors will be increased by treating those infected. Unfortunately, effectiveness of etiological treatment for these primary prevention indications remains unknown, although it can be assumed to be at least equal to seronegativization rates observed in available studies. Another strategy would be the development of a treatment that can be administered to pregnant women, such as is used for HIV infection, to avoid congenital transmission during pregnancy. However, safety information on these drugs would be necessary for this strategy, and this is not currently available.

Etiological treatment in case of accidents with material contaminated with parasites or blood samples of patients infected with *T. cruzi* could also be considered as an indication for primary prevention. Actually, the treatment is not strictly a prophylaxis because it is not possible to avoid the infections, but the infection can be aborted immediately after accidents with a timely treatment to get an appropriate concentration of specific drugs (recommendation (B), and evidence III) [33].

#### 3.3.2. Secondary Prevention Level

If prevention activities cannot avoid infection in children, the cure of infected children is still possible by prescribing etiological treatment [10–22]. In this regard, etiological treatment is indicated when damages from cardiac or digestive disease are not strongly present in these children. This is the best opportunity to get seronegatization and avoid disease, thus preserving social, mental, and physical health into adulthood [63, 64]. 

A national control program has been incorporated progressively in several Latin-American countries. It has consisted of the screening of child populations as a regular strategy to offer opportunities for diagnosis and treatment (recommendation (A), and evidence Type I) [65], as well as timely diagnosis and treatment of children born with congenital infection (recommendation (A), and evidence Type II). The positive effect of curing children detected by serological screening must be assessed by taking into account patterns of disease transmission, evolution, and a calculation of the burden of disease attributable to Chagas disease, in order to analyze the usefulness of serology as an indicator of the action against the vector. 

Other indication for etiological treatment in secondary prevention is to avoid reactivation of a chronic infection. Immunosuppression due to immunosuppressive therapies [36] or HIV/AIDS [52] increases the risk of reactivation in patients with chronic infection. Although the effectiveness of etiological treatment for the clinical control of episodes of reactivation has been proven, it is necessary to gather evidence as to whether preventive treatment is effective in patients with no signs of clinical reactivation and with abnormal immunological parameters [66]. In this regard, some protocols recommend the treatment of organ donors infected with* T. cruzi* in order to reduce risk of transmission by transplant [67]. In this case, the treatment should be considered an act of primary prevention (recommendation (A), and evidence Type II).

#### 3.3.3. Tertiary Prevention Level

The use of etiological treatment against *T. cruzi* infection in order to reduce the negative impact of established disease is under evaluation through two randomized clinical trials, which assess the efficacy in patients with cardiac disease [68–70]. These trials are appraising the efficacy of benznidazole for preventing progression of cardiac disease.

Several observational studies have been published showing effects of etiological treatment in patients infected with *T. cruzi*, on prevention of the progression of chronic chagasic cardiomyopathy [18, 27, 41, 43, 71]. These studies reached quality of evidence Type II, providing strength of recommendation (B) and (C). The prognosis of patients with heart failure or advanced stages of Chagas' cardiomyopathy is poor [72], but similar to others that develop heart failure for other reasons. Since the disease is chronic and heart damage develops over decades, it is very important to recognize factors that are determinant of disease progression in the early stages [73]. Etiological treatment should be considered as a protective factor in the model of physiopathology of Chagas' cardiomyopathy.

As mentioned above, the effectiveness of etiological treatment for the control of episodes of reactivation has been proven, showing recovery of severe manifestations of reactivation such as meningoencephalitis, myocarditis, and panniculitis [36, 52].

## 4. Discussion

Recommendations for appropriate care of patients are increasing, putting emphasis on the care of patients in the primary health care system, the use of other levels of care when necessary [74–76], and the incorporation of psychological aspects into care [63, 64]. In this context, it is important to consider the available evidence about etiological treatment, and to maintain the perspective of etiological treatment as a public health tool in multiple levels of prevention, along with other interventions available for Chagas disease control and treatment.

In Chagas disease, the best examples of primary prevention are vectorial control (based on surveillance) and control of blood and organ donors. However, etiological treatment has an important role in primary prevention and has to be considered a key element on among other strategies of the Chagas disease control programs.

The best example of secondary prevention in Chagas disease is the control of congenital transmission, and the diagnosis of infection in children (defined as chronic recent infection) or young-adult patients in chronic phase without clinical manifestations (sign and/or symptom) [77].

Use of etiological treatment for tertiary prevention in Chagas disease is currently supported by recommendation levels (B) and (C), when given in addition to complementary therapies in patients with cardiac disease to reduce the clinical progression of the disease. For instance, cardiac transplant is a procedure that has been applied and has demonstrated clinical benefit in some patients with terminal heart failure [78]. Stem cell transplant is a new therapy applied to produce cardiac regeneration through distinction or increase heart myocytes or neovascular proliferation in patients in the final stage of congestive heart failure [79–81], but the results are still insufficient on Chagas disease, and there is no consensus about its efficacy [80].

Regarding quaternary prevention, a national policy of etiological treatment of infected people should be considered as an activity. This approach has been utilized by several Latin-American countries in the last decades.

The assessment of effect of treatment against *T. cruzi* infection requires a clear understanding about the combination of variables to an appropriate interpretation of results to appraise. Among others, the main variables are the tools used as indicators (parasitological, molecular, and serological tests), the phase of infection (acute or chronic) that the patient is undergoing when he/she was treated, and the time elapsed between treatment and the application of the test to assess efficacy/failure.

The ideal assessment of response to specific treatment is the detection of free parasites in the patient's blood [82] or tissues [83], which permits clear observation of failure of treatment.

Only limited methods are available for assessing the efficacy of treatment. It is also necessary to validate new tools to confirm cure or failure in a timely manner after a full course of treatment has been given during the chronic phase, and studies are ongoing to validate PCR and standardized and validate qPCR.

If persistence of the parasite is identified, after verifying if the drug was taken correctly, it is necessary to consider the possibility that the parasite has developed resistance [84, 85]. Possible regional differences (host, *T. cruzi* strain, etc.), have also been described [10, 22], but more observation is needed to confirm this hypothesis.

After etiologic treatment, even in cured patients, the antibodies may remain detectable in sera for a long term (for years) until they become negative. Because of this phenomenon, it would be necessary to delve into the clinical history of patients with reactive serology, asking the question “did he/she receive treatment in the past?" When given an affirmative answer, the serological test has a limited value, because we must consider whether this reactivity is reflecting an active infection or if the patient was cured and he/she is becoming negative.

Current recommendations have put the bulk of the diagnostic and treatment responsibility on the primary health care system. Yet the management of infected patients has some basic limitations, but several researches are looking for solutions.

(a) Current drugs are able to cure infection (or prevent disease) in adult patients during the chronic phase, which is when the first contact is made with most infected patients, and clinical trials are finishing or ongoing to demonstrate effects of conventional treatment on this population [69, 70].

(b) New pediatric presentation of benznidazole is under evaluation to eliminate infection in newborns, and children with recent chronic infection [86]. Most new cases are, in fact, newborns with congenital infection.

Overall, the priorities in Chagas disease research should be to produce new drugs providing a shorter treatment course with fewer side effects, and to devise pediatric formulas. Some strategies, such as testing old drugs for extending current prescriptions, screening new compounds, testing drugs developed for other prescription such as pozanonzole, or developing new compounds are being used (Clinical Trial for the Treatment of Chronic Chagas Disease with Posaconazole and Benznidazole; NCT01162967) [69, 86]. Associations of compounds with different mechanisms of action have been mentioned as another way to look for new treatment alternatives [87].

Based on current disease understanding during chronic phase of infection, there is consensus that every patient infected with *T. cruzi must be* (children) or *should be* treated (adults). Treatment can cure infection and reduce or prevent the progression to the Chagas-related heart disease/cardiomyopathy. The current evidence of benefits and limitations of etiological treatment, based on clinical and implementation research, serve to prioritize strategies in primary health care, focusing on completing the scheme of treatment, rather than demonstrating serological negativization.

To incorporate etiological treatment as a public health strategy which is useful at the primary, secondary, and tertiary prevention is essential to reduce burden of the disease and to eliminate Chagas disease as a public health issue.

## Figures and Tables

**Table 1:** Indication of treatment against *Trypanosoma cruzi* infection based on different levels of quality of evidences and tools to assess efficacy or failure.

Indication (strength of the recommendation, and level of evidence)	Drug	Efficacy^†^	Time elapsed	Failure^‡^	Comments
Acute phase: vector transmission ((A) I) [10] Congenital transmission ((A) II) [11–15]	Bz, Nftx	65–100%	8 months or more	5%	Medium term of followup to asses efficacy Good tolerance

Early chronic phase (children) ((A) I) [16–22]	Bz	50–70%	3–15 years	5%	Most of the cases were children under 12 yo. Long term of followup to asses efficacy Good tolerance Different response for *T. cruzi* lineage I and II Some resistant clones were observed

Late chronic phase (adults, indeterminate, cardiac/digestive/other diseases) ((B) II; (C) II) [7, 18, 23–31]	Bz, Nftx	30%	>20 years	10%	Long term of followup Frequent side effects Efficacy to prevent evolution is under research Moderate-bad tolerance Different response for *T. cruzi* lineage I and II Some resistant clones were observed

Pregnant ((E) III) [32, 33]	NA	NA	NA	NA	Some accidental or necessary treatment during pregnant with acute phase did not show damaging effect in the child Treatment of pregnant women is currently not recommended [7, 9]

Immunocompromised (i.e., HIV, Transplant, other) ((A) II) [34–40]	Bz, Nftx	ND	ND	<5%	Etiological treatment aborts severe forms of reactivation as meningoencephalitis, myocarditis, panniculitis, and so forth, Good response No evidence about prophylaxis. Under research

Accidents ((B) III) [33]	Bz, Nftx	NA	NA	NA	10–15 days treatment immediately after accidents avoid infection

^†^Maximum rate of seronegativization.

^‡^Maximum rate of positive parasitologic test after treatment.

Bz: benznidazole, NA: not applicable, ND: no data, Nftx: nifurtimox.
